# Is Mindfulness the Common Ground Between Mental Toughness and Self-Compassion in Student Athletes? A Cross-Sectional Study

**DOI:** 10.3390/ejihpe15060095

**Published:** 2025-05-31

**Authors:** Zacharias Papadakis, Shana M. Walsh, Grant B. Morgan, Paul J. Deal, Andreas Stamatis

**Affiliations:** 1 Human Performance Laboratory, Department of Health Promotion and Clinical Practice, Barry University, Miami Shores, FL 33161, USA; zpapadakis@barry.edu; 2 School of Education, Peru State College, Peru, NE 68421, USA; swalsh@peru.edu; 3 Department of Educational Psychology, Baylor University, Waco, TX 76798, USA; grant_morgan@baylor.edu; 4 Department of Counselor Education, State University of New York at Plattsburgh, Plattsburgh, NY 12901, USA; pdeal001@plattsburgh.edu; 5 Department of Health & Sport Sciences, University of Louisville, Louisville, KY 40292, USA; 6 Sports Medicine Institute, University of Louisville Health, Louisville, KY 40208, USA

**Keywords:** positive psychology, NCAA, NAIA, mental toughness, self-compassion, mindfulness, collegiate athletics, sport psychology

## Abstract

This study interrogates whether mental toughness (MT) and self-compassion (SC)—historically framed as oppositional constructs—can coexist synergistically among NCAA Division II, III, and NAIA collegiate athletes, with mindfulness as a hypothesized mediator. A cross-sectional survey of 396 participants (mean age: 19.8 yrs ± 1.9 SD; females: 51%), revealed a robust MT–SC correlation (r = 0.46), which attenuated to 0.31 when mindfulness was modeled, signaling its role as a partial mediator. Hierarchical regression controlling for sex showed that MT and sex together explained 22% of the SC variance (ΔR^2^ = 0.22, *p* < 0.001). Adding mindfulness increased the total explained variance to 39% (ΔR^2^ = 0.17, *p* < 0.001). Females scored slightly lower on SC (β = –0.14, SE = 0.05, *p* = 0.008). Sobel testing confirmed significant partial mediation (Z = 7.22, *p* < 0.001), with mindfulness explaining 33% of MT’s total effect on SC. Mindfulness-based interventions that exploit athletes’ intrinsic attentional resources can simultaneously enhance mental toughness and self-compassion. By reconciling performance-oriented rigor with resilient self-regard, such strategies hold promise for athletes operating at diverse competitive levels.

## 1. Introduction

Mental toughness (MT) is widely regarded as a cornerstone of athletic success, denoting a constellation of adaptive psychological attributes that enable athletes to maintain high standards of training and competition despite adversity (Jones et al., 2007). Although definitions vary, most agree that it refers to a set of positive attributes that include effective coping strategies in the face of adversity and an ability to train and compete at consistent and maximal levels of performance (Gucciardi et al., 2015). Although typically celebrated, MT also carries potential liabilities: excessive self-criticism, overtraining, and diminished adherence to rehabilitation protocols have all been linked to its more rigid expressions (Jaeschke et al., 2016). Consequently, the pursuit of improving MT has drawn considerable attention from coaches, sports psychologists, tactical athletes, and athletes aiming to enhance performance while mitigating its darker side (Stamatis et al., 2020b, 2021, 2024).

Self-compassion (SC) has emerged as one such strategy, drawing interest as a potentially coping resource in sports. Research highlights the potential of self-compassion as a beneficial psychological tool for athletes, countering the common belief that self-criticism is necessary for performance enhancement. It is suggested that self-compassion can lead to significant improvements in athletes’ mental health and performance perceptions (Kuchar et al., 2023). Typically, SC encompasses three inter-related components: (1) self-kindness (e.g., being understanding to oneself versus being overly critical or judgmental), (2) common humanity (e.g., acknowledgment that others have similar experiences), and (3) mindfulness (e.g., awareness and balance of one’s thoughts and feelings without becoming overly reactive) (K. Neff, 2003). While SC is not yet thoroughly defined in sport-specific contexts, the consensus is that SC helps athletes face adversity and pain without excessive self-criticism or rumination, ultimately supporting emotional well-being (Leary et al., 2007). A recent review identified quantitative evidence that SC benefits athletes’ well-being and coping with challenges; moreover, there is no evidence that SC hinders performance. Some athletes, however, worry that SC could reduce the motivation for self-improvement (Röthlin et al., 2019). Thus, more empirical work is needed to clarify SC’s role and to understand how athletes perceive it.

### 1.1. Mental Toughness and Self-Compassion in Sport

At first glance, MT and SC may seem to represent divergent approaches to handling adversity. Regarding MT, it encourages pushing through discomfort and persisting despite challenges, potentially involving stern self-assessment (Gucciardi et al., 2009). From a self-regulation standpoint, MT embodies a higher-order capacity for goal-directed control over thoughts, emotions, and behavior (Carver & Scheier, 2000). Athletes who score high on MT subsequently display superior emotion-regulation skills and lower stress at a season’s end, with adaptive regulation mediating these effects (Crawford et al., 2023). Conservation of Resources theory further posits that personal resources accumulate in “caravans”, such that the possession of one resource increases the likelihood of gaining others (Holmgreen et al., 2017). By contrast, SC, defined by mindful, kind, and balanced self-responding, constitutes a downstream regulatory resource that can be built on the cognitive control and confidence afforded by MT. As such, it advocates for kind self-treatment and nonjudgmental acknowledgment of difficulties (K. Neff, 2003). Yet preliminary research suggests these constructs may coexist. Wilson et al., in a qualitative study of seven elite Canadian women athletes, found both MT and SC were critical for coping with sport-related adversity (Wilson et al., 2019). The athletes saw SC as compatible with MT and even integral to developing it (Mosewich et al., 2014). Interestingly, mindfulness, a component of SC, emerged as crucial to balancing the two (Gardner & Moore, 2012). The authors coined the term “the zipper effect” to illustrate how athletes relied on MT during rigorous training and competition phases and on SC during recovery, tapering periods, and even between attempts within a single event (Kellmann et al., 2018; Wilson et al., 2019). The “zipper effect” describes a dynamic, context-dependent interplay in which athletes alternate between MT-dominant and SC-dominant modes to sustain both performance and well-being (Wilson et al., 2019). Qualitative data show three complementary mechanisms: first, temporal sequencing: during high-intensity training or critical competition moments, MT enables focus, confidence, and persistence, whereas immediately afterwards, SC curbs rumination and promotes physiological and psychological recovery (Kellmann et al., 2018); second, situational dependence: athletes report switching to SC when an error, injury/niggle, or coach feedback threatens self-image and then “zipping” back to MT once equilibrium is restored (Mosewich et al., 2013, 2014); and third, mindfulness functions as a regulatory nexus: through cultivated meta-awareness, athletes can disengage from overly stringent self-evaluation and re-engage in self-compassion, thereby averting psychological resource depletion and sustaining adaptive performance (Gardner & Moore, 2012). This pattern fits Conservation of Resources theory: MT provides an initial resource caravan that enables the acquisition of the downstream resource of SC (Holmgreen et al., 2017). Moreover, this pattern is corroborated quantitatively by positive MT–SC correlations across competitive levels (Mahmoud & Sahar, 2019; Maranan & Lopez, 2021; Stamatis et al., 2021). Consistent with these theoretical and empirical insights, we conceptualize MT as an antecedent that predicts greater SC, a premise we test in the current mediation model.

### 1.2. Mindfulness in Sport

Mindfulness is frequently defined as focusing attention on the present moment with curiosity, openness, and acceptance (Bishop et al., 2004). In the context of Wilson et al.’s study, mindfulness allowed athletes to maintain attention during training or competition and to acknowledge feelings or setbacks without dwelling on them (Wilson et al., 2019). Such present-focused attention aligns with findings that mindfulness may reduce rumination, improve emotion regulation (Josefsson et al., 2017), increase flow states, enhance self-rated performance, and reduce worry (Glass et al., 2019). Mindfulness functions as an integrative mechanism that translates the goal-directed regulation characteristic of MT into the equitable, self-compassionate responding emblematic of SC. First, athletes exhibiting elevated MT demonstrate superior attentional control and enhanced emotional stability when confronted with stress (Crawford et al., 2023). These capacities provide a robust substrate for the cultivation of present-centered, nonreactive awareness, commonly termed dispositional mindfulness (Bishop et al., 2004). Second, mindful meta-awareness allows athletes to decenter from harsh self-evaluation, notice suffering without over-identification, and appraise setbacks as transient challenges rather than threats (Gardner & Moore, 2012). Finally, the Mindful Self-Compassion model posits that such balanced awareness is the necessary precursor to extending kindness and common humanity toward oneself (K. Neff, 2003; K. D. Neff & Dahm, 2015). Empirically, mindfulness partially mediates positive associations between MT and adaptive outcomes in NCAA athletes (N = 542), with the mindfulness facet of the Self-Compassion Scale showing the strongest link to MT (Stamatis et al., 2020a). Qualitative investigations corroborate these findings, indicating that mindfulness is pivotal for the development and maintenance of both MT and SC among elite female athletes (Wilson et al., 2019). Guided by this evidence, we position mindfulness as a mediating mechanism through which MT predicts greater SC in the current model.

Although SC includes a mindfulness component, SC’s mindfulness pertains primarily to acknowledging negative experiences with balance rather than avoidance or over-identification (K. D. Neff & Dahm, 2015). Mindfulness as a standalone construct is broader, encompassing awareness of any experience: positive, negative, or neutral (Bishop et al., 2004; K. D. Neff & Dahm, 2015). Thus, mindfulness and SC overlap but differ in scope. SC focuses on the athlete enduring an event, while mindfulness focuses on the event itself and one’s reaction to it (K. D. Neff & Dahm, 2015).

### 1.3. Measuring Relationships Between MT, SC, and Mindfulness

Wilson et al. used a qualitative approach with elite women athletes, demonstrating that mindfulness underpinned the MT–SC relationship (Wilson et al., 2019). Stamatis et al. (2020a) further supported these findings quantitatively, surveying 542 NCAA athletes (across Divisions I, II, and III) and finding that MT and SC were related, with mindfulness acting as a connector. Therefore, including mindfulness skills in programs targeting MT and SC was recommended.

To build on this evidence, more research is needed among diverse athlete populations. NAIA athletes, for example, represent a large group (~77,000 participants across 250 institutions in the U.S.) yet remain under-represented in research (NAIA, 2025). NAIA-level competition often parallels NCAA DII and DIII in institutional size, enrollment, and athletic scholarship philosophy, often overlooked in research and remained under-represented in empirical work (Huffman, 2013; Nite et al., 2016). Expanding the investigation of MT, SC, and mindfulness to NAIA athletes can strengthen the external validity of earlier findings.

For example, mindfulness was found to correlate positively with psychological skills and mental toughness, suggesting that athletes with higher mindfulness levels tend to exhibit better psychological skills and greater mental toughness. This relationship underscores the potential of mindfulness as a tool for enhancing sports performance through psychological resilience and skill development (Wu et al., 2021). Unlike earlier investigations that considered mindfulness as a trained skill (Kabat-Zinn, 2003), our study focused on dispositional mindfulness (Brown & Ryan, 2003). While dispositional mindfulness reflects an inherent trait, mindfulness-based interventions can help athletes optimize how they apply this trait during training, competition, and recovery (Gardner & Moore, 2012). By using a quantitative methodology across different competitive levels and examining trait mindfulness, we gain insight into how these constructs interact naturally among a broader population of collegiate athletes (Gucciardi et al., 2015).

The present study aimed to determine if the MT–SC relationship, as previously identified among elite women athletes (Wilson et al., 2019) and NCAA athletes (Stamatis et al., 2020a), would also appear in NCAA DII, DIII, and NAIA athletes. We hypothesized that MT would correlate positively with SC, and that mindfulness—specifically, the mindfulness subscale from the Self-Compassion Scale—would mediate this relationship (K. Neff, 2003). Demonstrating such a linkage would justify mindfulness-based interventions aimed at simultaneously bolstering MT and fostering SC, thereby optimizing performance and well-being in a broader collegiate athlete population (Gardner & Moore, 2012; Kabat-Zinn, 2003). Such findings could inform coaching strategies, mental skills training, and program development aimed at enhancing performance and well-being.

## 2. Materials and Methods

### 2.1. Study Design and Ethical Considerations

This one-month-long cross-sectional study examined relationships between MT, SC, and mindfulness in collegiate athletes at DII, DIII, and NAIA levels. The methods and reporting adhere to the Strengthening the Reporting of Observational Studies in Epidemiology (STROBE) guidelines (Von Elm et al., 2007). Institutional Review Board (IRB) approval was obtained from [REDACTED] (number: [REDACTED]; date: [REDACTED]). Participants provided informed consent electronically before starting the online survey, which was anonymous and offered no incentives.

Surveys were distributed via Qualtrics, and up to three weekly email reminders were sent, with no further follow-up after that. The opening screen of the Qualtrics survey presented the study’s purpose, eligibility criteria, and guaranteed anonymity and required active-choice consent (i.e., voluntary participation) before entry. Recruitment e-mails, disseminated through the co-authors to different institutions, offered no incentives, thereby limiting volunteer bias linked to reward. To prevent duplicate submissions, we activated Qualtrics’ related option, and we restricted entries to a single IP address. Three gating questions verified roster status, age (≥18 years), and competition level; any failure terminated participation automatically. Within each scale block, items were randomized, and forced-response settings ensured complete data for the key variables: mental toughness, self-compassion, and mindfulness (Eysenbach, 2004).

### 2.2. Participants and Recruitment

A convenience sample of 396 undergraduate student athletes from three U.S. colleges/universities (in the Southeastern, Midwestern, and Northeastern regions) participated. The sample included 191 (48%) DIII athletes, 122 (31%) DII athletes, and 83 (21%) NAIA athletes. Approximately half were female (51%), and half were male (49%). The mean age was 19.8 years (SD = 1.9). The majority identified as White (n = 276; 80%), with the next largest groups identifying as Hispanic/Latinx (n = 77; 20%) and African American (n = 28; 7%) and fewer than 2% identifying with another race/ethnicity.

Participants (i.e., all student athletes from all the available sports offered based on their respective division) were recruited via emails sent to their institutional student email accounts. Coaches and athletic directors encouraged participation. Participants received an initial email followed by up to three weekly reminders if they did not complete the survey.

### 2.3. Measures

Sociodemographic/background characteristics: Participants answered questions about their demographic information and general background in sport.

Mental toughness (MT): MT was measured using the Mental Toughness Index (MTI) developed by Gucciardi et al. (Gucciardi et al., 2015). The MTI includes 8 items rated from 1 (true 100% of the time) to 7 (false 100% of the time). The items assess the ability to overcome adversity, control emotions, and maintain attentional focus. The MTI was selected because it was purposely built for sport contexts (Gucciardi et al., 2015) and now enjoys a substantial body of evidence supporting its reliability, factorial integrity, cross-cultural invariance, and practical sensitivity (Stamatis et al., 2021, 2022, 2023). The coefficient alpha estimate in this sample was 0.88.

Self-compassion (SC): SC was assessed with the 26-item Self-Compassion Scale (SCS) by K. Neff (2003), rated from 1 (almost never) to 5 (almost always). The SCS comprises six subscales: self-kindness, self-judgment, common humanity, isolation, mindfulness, and over-identification. The total SC score reflects the balance of these components. There is a compelling body of reliability and validity evidence supporting the use of the SCS (K. Neff, 2003), and it has been used previously with athletes (Mosewich et al., 2013). The coefficient alpha estimate in this sample was 0.90.

For this study, we (a) computed both the total self-compassion score and (b) isolated the mindfulness subscale, treating the latter as a separate variable. Because mindfulness is conceptualized as a stable, trait-like disposition, we retained Bishop et al.’s two-component framework, which offers construct equivalence across the 27 different sports represented in our sample and preserves replication continuity with prior MT–SC research (Bishop et al., 2004). Sport-specific mindfulness scales were not used, as they often suffer metric-invariance problems when applied to heterogeneous, multi-sport samples.

### 2.4. Data Analysis

**Figure 1:**
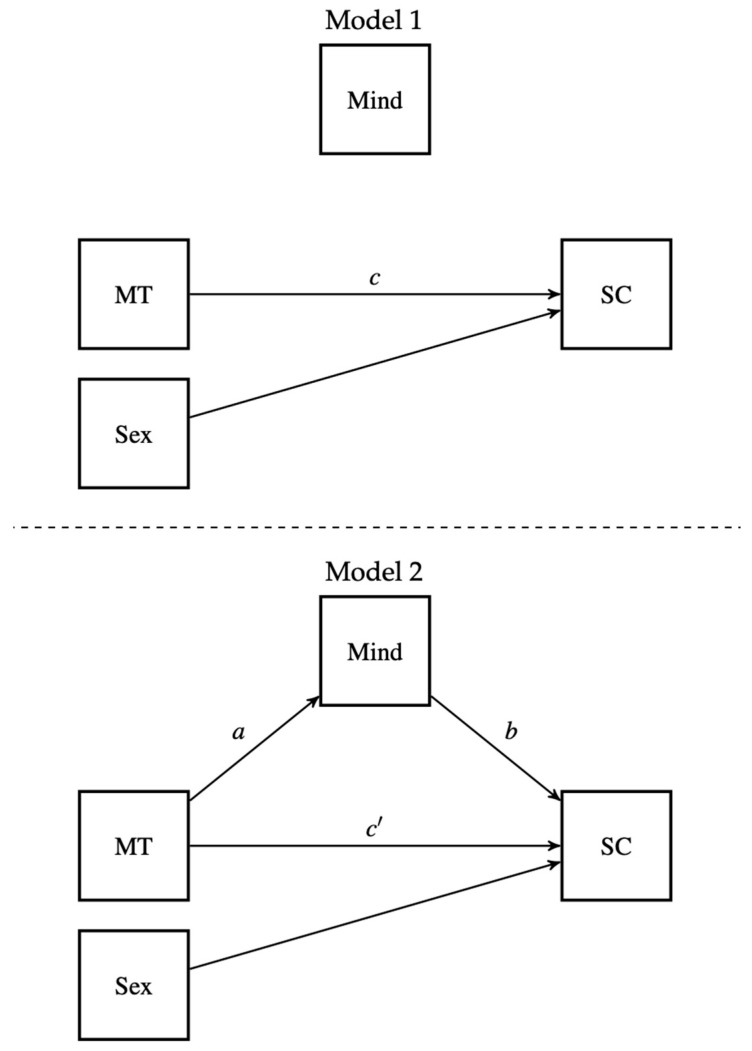
Path diagrams for estimated models. Note: sex (not displayed) was included as a covariate predicting SC.

We computed descriptive statistics for all primary variables and examined their distributional characteristics, ensuring that the conditions of normality, heteroscedasticity, and multicollinearity were tenable. Normality was evaluated by reviewing the skewness and kurtosis estimates (i.e., <|2.0|; Kline, 2015) and the Q-Q plot of the observed values against the expected quantiles of the normal distribution. Heteroscedasticity was evaluated by reviewing the plot of standardized residuals. Multicollinearity was evaluated be comparing the variance inflation factor (VIF) against a maximum cutoff of 5.0 (Sheather, 2009). It should be noted that the mindfulness subscale was extracted from the Self-Compassion Scale prior to conducting the analyses (VIFs for all predictors < 1.3 after removal; Table 2) to avoid potential multicollinearity. Zero-order correlations tested the MT–SC relationship. Next, we introduced mindfulness to assess its potential mediating effect. A classical mediation analysis was conducted via sequential linear regressions, where SC was regressed on MT alone (i.e., Model 1) and then on MT plus mindfulness (Model 2). Sex (0 = male, 1 = female) was entered as a covariate in both regression steps. The indirect effect was calculated using the product of coefficients method, and a Sobel test assessed statistical significance. The models are shown in Figure 1. All analyses were conducted using SPSS 26.0 software. It should be noted that one respondent skipped one item, and another respondent skipped two items on the SC scale. For those two respondents, the mean of the SC scale was calculated out of the number of completed items (i.e., 24 and 25, respectively, out of 26). The pre-established maximum Type I error rate was set to 5%.

## 3. Results

### 3.1. Descriptive Statistics

Descriptive statistics are shown in Table 1. MT scores ranged from 10 to 56, with a mean of 45.8 (*SD* = 6.7, *sk* = −0.8, *ku* = 1.8). Mindfulness (1–5 scale) ranged from 1 to 5 (*M* = 3.4, *SD* = 0.8, *sk* = −0.0, *ku* = −0.3), and SC ranged from 1.4 to 4.9 (*M* = 3.1, *SD* = 0.6, *sk* = −0.1, *ku* = 0.2). No significant differences emerged in average MT, mindfulness, or SC scores by sex or division; however, regression analyses later revealed a small, adjusted sex effect.

### 3.2. Correlations and Regression Analyses

The zero-order correlation between MT and SC was 0.46 (*p* < 0.001), indicating that athletes with higher MT tended to have higher SC as well. After controlling for mindfulness, the MT–SC correlation decreased to 0.31 (*p* < 0.001), suggesting that mindfulness accounts for a portion of their shared variance.

**Figure 2:**
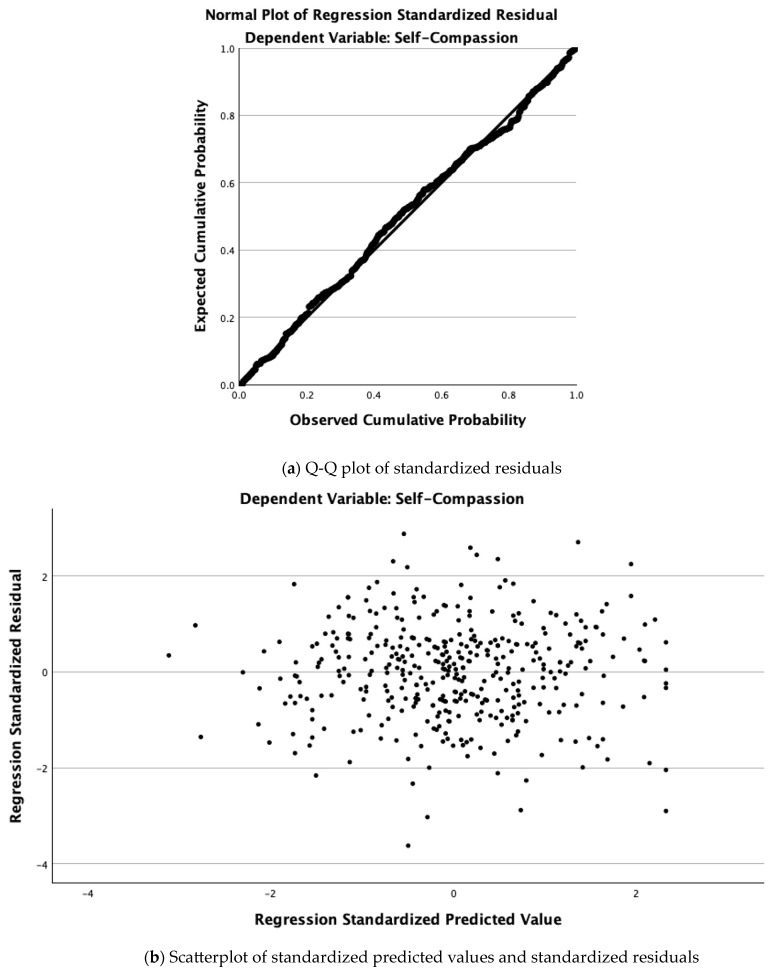
Diagnostic plots from Model 2.

When SC was regressed on MT and sex (Model 1), the two predictors explained 22% of the variance in SC (Table 2). MT was a positive predictor (β = 0.04, *p* < 0.001), whereas sex was negative (β = –0.16, *p* = 0.003), indicating that female athletes reported lower SC once MT was held constant. Adding mindfulness in Model 2 increased the explained variance to 39% (ΔR^2^ = 0.17). In the final model, mindfulness was the strongest predictor (β = 0.44, SE = 0.03, *p* < 0.001), MT retained a modest unique contribution (β = 0.25, SE = 0.00, *p* < 0.001), and sex remained a small but significant negative covariate (β = –0.14, SE = 0.05, *p* = 0.008). A one-point increase in mindfulness predicted a 0.35-point increase in SC, whereas being female predicted a 0.14-point decrease in SC after accounting for MT. The Q–Q plot of residuals and the residual-by-predicted plot are provided in Figure 2.

### 3.3. Mediation Analysis

The mediation analysis indicated that mindfulness partially mediated the relationship between MT and SC. The indirect effect (MT → mindfulness → SC) was 0.014 (0.04 × 0.35 = 0.014; Z = 5.46, CI95 = 0.01, 0.02), demonstrating that mindfulness significantly explains part of the MT–SC linkage.

## 4. Discussion

This study investigated whether the previously identified relationship between MT and SC, seen in elite athletes (Wilson et al., 2019) and NCAA athletes (Stamatis et al., 2020a), also exists across DII, DIII, and NAIA athletes, and whether mindfulness mediates this link. It was hypothesized that MT would correlate positively with SC, and that mindfulness—specifically, the mindfulness subscale from the Self-Compassion Scale—would mediate this relationship. Our results supported our hypothesis. We found that MT and SC are positively related, and when mindfulness is accounted for, their association remains but is reduced, indicating that mindfulness contributes substantially to their interplay (Baron & Kenny, 1986; Preacher & Hayes, 2008).

### 4.1. MT and SC Coexistence

Although MT and SC may appear conceptually dissimilar, our findings and previous research suggest they can coexist within the same individual (Mosewich et al., 2014). Athletes who are mentally tough (purposeful, efficient, and flexible) can also be self-compassionate, treating themselves kindly when facing setbacks (K. Neff, 2003). Wilson et al.’s qualitative work described a temporal and contextual interplay: athletes relied on MT during training and competition but shifted toward SC during tapering, recovery, or between attempts (Wilson et al., 2019). Our quantitative findings echo this compatibility, suggesting these constructs may complement each other in dynamic ways.

It is noteworthy that while MT has sometimes been associated with traits that could impair well-being (e.g., self-criticism), SC’s emphasis on kindness and mindfulness may buffer these negative elements (K. Neff, 2003; K. D. Neff & Dahm, 2015). Considering mindfulness as a mediator provides insight: by fostering present-moment awareness (Bishop et al., 2004) and reducing excessive self-judgment, mindfulness may help integrate MT and SC, allowing athletes to push hard when needed and recover mentally without unnecessary self-punishment (Gardner & Moore, 2012).

### 4.2. Role of Mindfulness as a Mediator

Mindfulness functions not merely as an adjunct to MT and SC; rather, it constitutes the integrative fulcrum that unifies these constructs (Wilson et al., 2019), which harmonizes their interplay. While Baron and Kenny’s mediation framework anchors our analysis, the partial mediation effect hints at a nuanced synergy: mindfulness may enable athletes to toggle between relentless drive and compassionate recovery, a duality critical to peak performance (Baron & Kenny, 1986). Importantly, these findings suggest that while dispositional mindfulness may be a trait-like construct (Brown & Ryan, 2003)—and meta-analyses confirm that 4- to 12-week mindfulness programs produce small-to-moderate increases in dispositional scores (Donald et al., 2019)—mindfulness-based interventions can enhance athletes’ ability to apply this trait effectively in performance contexts. This aligns with research indicating that mindfulness training can reduce rumination and improve emotion regulation (Josefsson et al., 2017), enhance flow states (Glass et al., 2019), and improve overall well-being (Kabat-Zinn, 2003).

Mindfulness-based interventions are increasingly popular in sport psychology (Carraça et al., 2018). Programs like the Mindfulness–Acceptance–Commitment (MAC) approach, Acceptance and Commitment Therapy (ACT), and Mindful Sport Performance Enhancement (MSPE) have shown promise in improving mental states, focus, and performance among athletes (Carraça et al., 2018; Glass et al., 2019). Such interventions could serve as strategic methods to simultaneously bolster MT and SC.

### 4.3. Sex Differences and Sample Diversity

Although raw means did not differ by sex, multivariable analysis showed that female athletes reported slightly lower self-compassion (β = –0.14, SE = 0.05, *p* = 0.008) once MT and mindfulness were held constant. This residual effect accords with earlier evidence that women tend to experience greater self-criticism and rumination (K. Neff, 2003; Mosewich et al., 2014). While the magnitude was small (ΔR^2^ ≈ 0.02), the finding suggests that self-compassion content may need extra emphasis for female athletes, for example, by incorporating exercises that explicitly target self-kindness and cognitive-reframing strategies. Our inclusion of NAIA athletes, who are often neglected in research (Nite et al., 2016), broadens the generalizability of prior findings from elite women athletes (Wilson et al., 2019) and NCAA athletes (Stamatis et al., 2020a). The results suggest that the MT–SC–mindfulness relationship holds across various competition levels, supporting the external validity of these constructs and their interplay (Anderson-Cook, 2005). This more inclusive approach can guide sport psychologists and coaches working with diverse populations.

### 4.4. Conceptual and Practical Implications

Conceptually, this study adds to the growing body of literature suggesting that positive psychological constructs in sport do not exist in isolation (Seligman & Csikszentmihalyi, 2000). Mental toughness and SC can coexist and may be linked by mindfulness (Wilson et al., 2019). Practically, these findings encourage integrated mental training approaches. Coaches and practitioners might combine MT training (e.g., goal setting and persistence drills) (Gucciardi et al., 2009, 2015, 2016) with SC-related activities (e.g., guided self-reflection and reframing mistakes with kindness) (K. Neff, 2003) and mindfulness exercises (e.g., breathwork and body scans) to create a comprehensive mental conditioning program (Gardner & Moore, 2012).

By recognizing that mindfulness may help athletes navigate the tension between relentless effort (MT) and compassionate self-care (SC), professionals can design interventions that foster resilience without sacrificing well-being (Mosewich et al., 2013, 2014), particularly for female athletes, who exhibited a small self-compassion deficit after adjustment. For instance, mindfulness exercises taught during offseason or pre-competition phases could help athletes learn to switch seamlessly between pushing their limits and treating themselves kindly, depending on situational demands (Gucciardi et al., 2016; Wilson et al., 2019).

### 4.5. Limitations and Future Research

This study is not without limitations. Our sample, although robust, was a convenience sample and may not be fully representative of all student athletes at these competitive levels. The number of athletes from each division was unequal, potentially affecting the results (Field, 2018). Additionally, our cross-sectional design cannot establish causality or temporal ordering between MT, SC, and mindfulness (Maxwell & Cole, 2007). Future longitudinal or experimental research could clarify whether increases in mindfulness lead to simultaneous improvements in MT and SC (Kazdin, 2007).

We also did not deeply explore cultural nuances. Mindfulness, originally rooted in Eastern contemplative traditions, is often adapted in Western sport contexts with minimal reference to its cultural and philosophical origins (Roychowdhury et al., 2021). Researchers and practitioners should approach mindfulness interventions with cultural sensitivity and an awareness of these roots (Kabat-Zinn, 2003).

Future investigations should extend these findings across diverse athletic cohorts through longitudinal and experimental designs—for example, prospective cohort studies and randomized controlled trials—to clarify causal pathways among MT, SC, and mindfulness (Maxwell & Cole, 2007). In mediation testing, bias-corrected bootstrapped confidence intervals can supplant the Sobel test, providing more reliable estimates of indirect effects. Methodologically, researchers ought to employ culturally responsive protocols, recognizing mindfulness’s migration from Eastern contemplative traditions to Western sport contexts (Roychowdhury et al., 2021). Comparative trials could determine whether targeted mindfulness programs differentially augment MT and SC (Josefsson et al., 2017) or whether SC-focused interventions elevate MT indirectly via enhanced mindfulness (Mosewich et al., 2014). Moderator analyses considering the sport type, competition level, and cultural background will refine our understanding of situational contingencies. Finally, because the present study treated sex as binary, future work should incorporate gender-diverse samples and test sex/gender as potential moderators to ensure findings generalize across the full athlete spectrum. Integrated research along these lines will clarify how cultivating MT, SC, and mindfulness can optimize performance while safeguarding psychological health (Seligman & Csikszentmihalyi, 2000).

## 5. Conclusions

This study provides quantitative evidence that MT and SC are not mutually exclusive in collegiate athletes across competitive tiers; rather, they coexist and are linked through mindfulness, which emerged as a partial mediator of their association (Baron & Kenny, 1986; Stamatis et al., 2020a, 2020b, 2024; Wilson et al., 2019). Although effect sizes were small, female athletes exhibited marginally lower SC after accounting for MT and mindfulness, signaling a potential focus for targeted interventions (Kabat-Zinn, 2003). These findings extend earlier work confined to elite samples, reinforce resource-based and mindful-self-compassion theories, and outline an empirical foundation for integrated mental-skills programs that cultivate MT, mindfulness, and SC in concert (Gardner & Moore, 2012; Huffman, 2013; Nite et al., 2016). In summary, cultivating this triad may enable athletes to pursue peak performance while safeguarding psychological well-being.

## Figures and Tables

**Table 1:** Descriptive statistics for included variables.

Sample	Mental Toughness		Mindfulness		Self-Compassion	
	*M*	*SD*	*M*	*SD*	*M*	*SD*
Overall	45.8	6.7	3.4	0.8	3.1	0.6
Sex						
Female	44.0	6.0	3.2	0.7	2.9	0.6
Male	47.6	6.9	3.5	0.8	3.3	0.5
Division						
DII	46.7	6.3	3.4	0.8	3.1	0.5
DIII	45.4	6.8	3.4	0.7	3.2	0.6
NAIA	45.4	7.1	3.2	0.8	2.9	0.6

Note. *M* = mean, *SD* = standard deviation.

**Table 2:** Regression model results.

			95% Confidence Interval				
Variable	Coefficient	SE	*Lower*	*Upper*	β	$R^{2}$ (CI95%)	VIF
Model 1							
Mental toughness	0.04 ^†^	0.00	0.01	0.03	0.04	0.22 (0.15, 0.29)	1.08
Female	−0.19	0.06	−0.31	−0.08	−0.16		
Model 2							
Mindfulness	0.35	0.03	0.28	0.41	0.44	0.39 (0.32, 0.46)	1.17
Mental toughness	0.02	0.00	0.02	0.03	0.25		1.22
Female	−0.14	0.05	−0.24	−0.05	−0.12		1.09
Indirect Effect							
	***Estimate***	***Z***	***Lower***	***Upper***	***p***		
MT × mindfulness	0.014	7.22	0.01	0.02	<0.001		

^†^ = total effect of MT on SC. Note: SE = standard error, VIF = variance inflation factor, and MT = mental toughness. Sex coded: 1 = female; 0 = male.

## Data Availability

Requests to access the datasets should be directed to the corresponding author.
